# Association between a novel dietary lipophilic index (LI) with metabolic phenotypes in a community-based study in Tabriz- Iran

**DOI:** 10.1186/s12902-020-00638-w

**Published:** 2020-10-27

**Authors:** Nika Soltani, Mahdieh Abbasalizad Farhangi, Leila Nikniaz, Mahsa Mahmoudinezhad

**Affiliations:** 1 Molecular Medicine Research Center, Tabriz University of Medical Sciences, Tabriz, Iran; 2 Drug Applied Research Center, Tabriz University of Medical Sciences, Tabriz, Iran; 3 Tabriz Health Services Management Research Center, Health Management and Safety Promotion Research Institute, Tabriz University of Medical Sciences, Tabriz, Iran

**Keywords:** Lipophilic index, Metabolic parameters, Metabolic syndrome, Dyslipidemia

## Abstract

**Background:**

Dietary fatty acids are important dietary determinants of metabolic disorders in human. However, it is important to develop an index that considers not only the amount of dietary fatty acids but also the structure, fluidity and melting point of them. In the current study we investigated the association between a novel dietary lipophilic index (LI) with metabolic profile and dyslipidemia in a community based study in Tabriz-Iran.

**Methods:**

Dietary data were collected using a validated, 79-food item, semi-quantitative food frequency questionnaire, and dietary LI was calculated. Anthropometric variables were measured and metabolic profile including blood sugar, serum lipids and liver enzymes were assessed. Metabolic syndrome was defined according to the adult treatment panel (ATP) III criteria.

**Results:**

The mean age of the participants was 42.53 ± 12.03 years and most of the participants were women. Mean of dietary LI was 34.99 ± 6.91. Higher dietary LI was associated with higher body mass index (BMI) (β = 0.17, *P* < 0.04), waist circumference (β = 0.18, *P* < 0.01) and systolic blood pressure (β = 0.27, *P* < 0.01). Also LI was increased with increasing waist circumference (0.001), low density lipoprotein cholesterol (LDL-C) (0.001), and negatively associated with high density lipoprotein cholesterol (HDL-C) (0.001).

**Conclusion:**

The novel dietary LI was considered as a useful tool in prediction of cardio-metabolic risk factors including general and central obesity, dyslipidemia and metabolic syndrome in a population-based study in Iran. Further researches in other disease and populations could highlight the application of this index in clinical settings.

## Background

Metabolic syndrome (MetS) consists of various metabolic abnormalities. The prevalence of MetS is approximately 25% in adults which is increasing in advanced ages. Lifestyle-related factors, particularly physical activity and dietary habits are important determinants of MetS. The risk of MetS is significantly lower in physically active, non-smoker, and normal weight individuals [1, 2]. Dietary fat is one of the most important contributing factors that affects metabolic profile and is involved in the progression of MetS. Numerous studies revealed the beneficial role of vegetable oils and polyunsaturated fatty acids in protection of metabolic abnormalities and these studies revealed the adverse effects of saturated fatty acids and animal fats on metabolic panel and progression of MetS [3–5]. Dietary fatty acids are categorized into several groups according to the presence and number of *double* bonds, length of the chain and their configuration—*cis* or *trans* [6]. Several studies have revealed the association between different kinds of fatty acids with components of MetS and dyslipidemia. A recent study in Iran evaluated the association between saturated and unsaturated fatty acids from different food groups and the risk of MetS. The result revealed a direct relationship between intake of saturated fatty acids from different food groups and MetS [7]. In another study by Freire RD et al. in a population-based study of Japanese Brazilians aged more than 30 years, individuals in the highest quintile of total dietary fat consumption were five times more likely to develop MetS compared with those in the lowest quintile [odds ratio (OR): 5.0 ([95% CI 1.58–16.00]; *P* < 0.005). While linoleic acid intake was inversely associated with MetS (OR: 0.50 [95% CI 0.26–0.98]; *P* < 0.05). Most of the studies have examined the role of only a limited number of isolated fatty acids; while in a usual diet, a combination of fatty acids are consumed with numerous synergistic or inhibitory actions; therefore, developing an index that contributes dietary pattern of fatty acids’ intake in the usual diet might give a realistic image of the human diet; recently, a new lipophilic index (LI) has been developed that is calculated as: the sum of the quantity of fatty acids consumed by an individual multiplied by melting point of each fatty acid then divided by the total grams of fat intake [8, 9]. It was used to determine the overall fluidity of the membrane that is dependent on all of the fatty acids constituting the membrane structure. Melting point is a characteristic of fatty acids that depends on the chain length and the degree of saturation [10]; therefore, long chain fatty acids have less fluidity. Increased membrane fluidity has various effects on health, including increase of high-density lipoproteins’ amounts [11], improved insulin binding [12], reduced insulin resistance [13] and reduced risk of hypertension [14]. To the best of our knowledge [11], no study has examined the association between MetS and its ingredients with dietary LI. Two studies have examined the association between dietary LI and risk of myocardial infarction (MI). In a case-control study in Costa Rica, Estefania Toledo et al. found that a higher dietary LI reduced fatty acid fluidity and had an important role in the etiology of coronary heart disease [9]. In another study Qing Liu et al. indicated that higher LI was associated with elevated risk of congestive heart disease (CHD) among postmenopausal women [15]. Considering the high prevalence of MetS and the lack of studies that have examined the association between MetS components and dietary LI, this study was conducted to determine the association between dietary LI and components of the MetS in a community-based study in Tabriz, Iran.

## Methods

### Participants

**Fig. 1:**
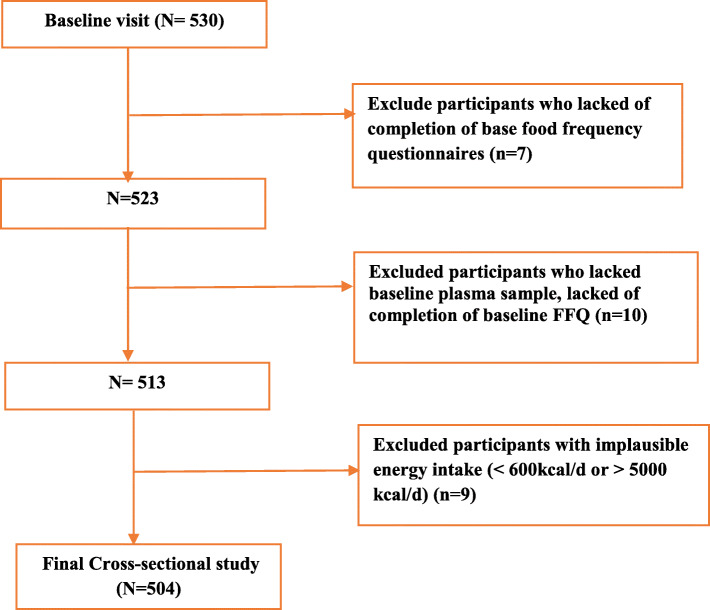
The flowchart of participants’ recruitment

This study is a part of Lifestyle Promotion Project (LPP), a cross sectional population based study that was conducted in East Azerbaijan (urban and regional parts)-Iran [16]. The sampling frame was defined by postal codes according to national post office and sampling method was the probability proportional to size multistage stratified cluster sampling method [17]. So the clusters were chosen based on postal code in the form of 10-digit code and the clusters included one to several blocks. Then, data collection began after determining clusters. In the following, houshold selection started at the cluster start point and continued due to right-hand side of each building. In total, 550 participants from 150 clusters were selected and one adult was randomly selected from each household. Forty-six participants were excluded due to incomplete demographic information and the final analysis was performed on 504 participants. The flowchart of participants’ recruitment is presented in Fig. 1. Inclusion criteria were as follow: willingness to participate in the study, apparently healthy individuals with age range of 18–64 years. Pregnant women or adults with severe chronic diseases requiring bed rest, physical and mental disability and the presence of communication barriers were excluded. Also, those with incomplete survey questionnaires or very low or high energy consumption were excluded. The research protocol was approved by the research undersecretary of Tabriz University of Medical Science (registration number: IR.TBZMED.REC.1397.333). A written informed consent was obtained from all of the individual participants included in the study. Participants were visited at their homes, then the information about their dietary intakes and health information, demographic characteristics, lifestyle and medical history, anthropometric measurements, and biological samples were collected.

### Dietary assessments and dietary lipophilic index (LI) calculation

**Fig. 2:**
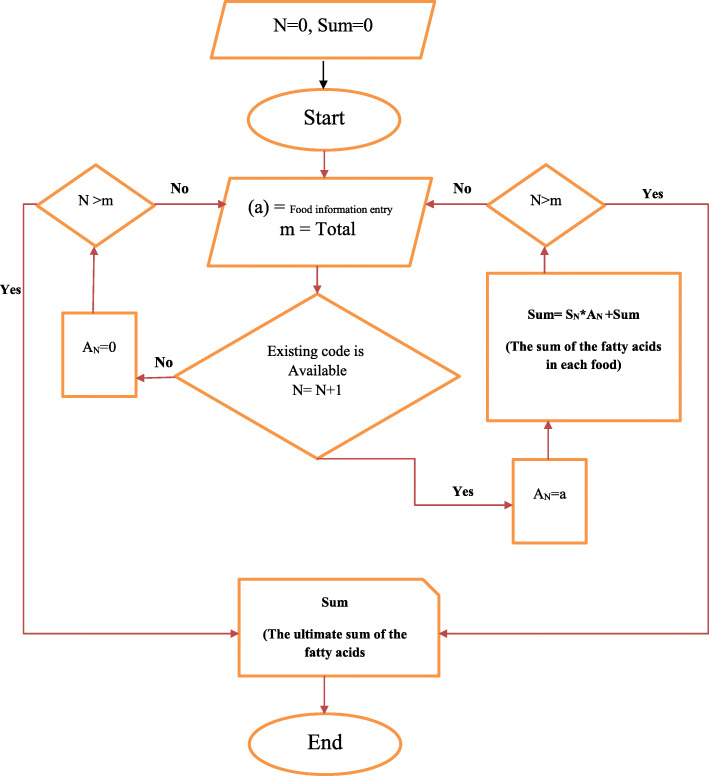
The flowchart of program written for estimation of individual dietary fatty acid content

Dietary pattern was evaluated using a validated 80 item food frequency questionnaire (FFQ) and its validity and reliability was assessed during the project [16, 18]. The amount of consumed food was reported by the individual and then converted to grams. Then, dietary intake of macro and micronutrients were estimated. The United States Department of Agriculture (USDA) National Nutrient Database for Standard Reference was used to estimate the consumed fatty acids in foods for each individual [19]. For estimation of individual dietary fatty acid intake, in the large number of foods with a great variation of fatty acids between different foods, a program in Excel was developed (Fig. 2). The program was written as macro programming codes written in Visual Basics for Applications (VBA). The dietary LI was calculated by summing the multiplication of each fatty acids intake (in grams) into its specific melting point (°C), divided to the sum of the consumed fatty acids [8]. Among forty nine fatty acids, melting points of 37 fatty acids were available and the information for the participants’ dietary fatty acids’ intake was available for thirty fatty acids. Information about the melting point and molecular weight was acquired from the Lipid Bank Database [10]. The LI calculation was performed as below:

$$ Dietary\  LI=\frac{\sum_K^i\ \left[ fatty\ acid\ (g)i\times melting\ point\ \left({}^{\circ}C\right)i\right]}{\sum_k^i fatty\ acid\ (g)i} $$

### The assessment of anthropometric variables and physical activity

All anthropometric measurements were performed by trained nutritionist. It means that, weight was measured using digital calibrated scales, with minimal clothes and without shoes, with an accuracy of 100 g. Height was measured by a standing stadiometer with an accuracy of 0.1 cm; body mass index (BMI) was calculated as weight divided by height squared (kg/m^2^). Waist circumference (WC) was measured with a 0.1 cm precision strap between the smallest area below the ribs and above the umbilicus; hip circumference (HC) was measured at the level of the greatest protrusion of the buttocks when the subject was standing erect with the feet together. Physical activity (PA) was calculated by multiplying the frequency, duration, and intensity of physical activity using International Physical Activity Questionnaire-Short Form (IPA-SF) [1, 13].

### Blood pressure and the assessment of metabolic profile

Blood pressure was measured after a 10-min resting using a mercury barometer on the left arm. Blood pressure was measured twice and the mean blood pressure was calculated and reported as the blood pressure of each person. According to the seventh report of the Joint National Committee on Prevention, Detection, Evaluation, and Treatment of High Blood Pressure (JNC-7), hypertension was defined as having systolic blood pressure (SBP) greater than or equal to 140 mmHg or diastolic blood pressure (DBP) greater than or equal to 90 mmHg. On the first visit, blood samples were taken from individuals and were stored at − 70 °C under nitrogen until analysis. Enzymatic colorimetric method was used for measuring the levels of serum high density lipoprotein concentrations (HDL-C), triglyceride (TG), low density lipoprotein cholesterol (LDL-C), total cholesterol (TC) and fasting blood sugar (FBS) using commercial kits (Pars Azmon, Tehran, Iran) with an automatic analyzer (Abbott, model Alcyon 300, USA). MetS components were assessed according to the adult treatment panel (ATP) III criteria including: abdominal obesity (waist circumference greater than 102 cm in men, and more than 88 cm in women), HDL-C < 40 mg/dl in men and HDL-C < 50 in women, TG ≥ 150 mg/dl, SBP ≥ 130 mmHg, DBP ≥ 85 mmHg and FBS ≥ 110 mg/dl. Dyslipidemia was defined as TC, LDL-C and TG values higher than 200, 130, 150 mg/dl respectively and serum HDL-C less than 40 and 50 mg/dl in men and women respectively. All biochemical assessments done by laboratory expert.

### Statistical analysis

Data analyses was done using Statistical Analysis System (SAS, v. 9.2, SAS Institute Inc., Cary, USA). Descriptive measures were used to check the normal distribution of data. The comparison of participants’ general characteristics in different tertiles of dietary LI was performed by one-way analysis of variance (ANOVA) or Kruskal-Wallis test for continuous and the χ^2^ test for categorized variables. Linear trends were analyzed using the Cochran-Armitage test. Pearson’s correlation coefficients was used to examine the correlations between continuous variables. Comparison of means or frequency for baseline characteristics and metabolic biochemical variables, adjusted by confounders, were tested using the multivariate analysis of covariance and regression analysis. *P*-values of less than 0.05 were considered as statistically significant.

## Results

Median LI values for the total population was 34.99 (6.91) mEq/day. Table 1 presents the comparison of baseline characteristics in different tertiles of dietary LI. Accordingly, mean LI in women was significantly lower than men (men: 36.59 ± 6.49; women: 3.73 ± 6.98; *P*-value = 0.01). Moreover, WC was significantly lower and BMI was significantly higher in the females compared to male participants (Table 1). Individuals in rural areas, with low educational attainment and higher central obesity had higher LI values (*P* < 0.05). Participants in the highest dietary LI tertiles were more likely to be male, married, living in rural areas and had poor educational attainment. Moreover, individuals in higher tertiles of LI had higher SBP, lower carbohydrate and higher protein and fat intake in their usual diet (P < 0.05). Accordingly, in comparison of biochemical variables between LI tertiles (Table 2), serum LDL and HDL values were significantly higher and lower among individuals of higher LI tertiles (*P* < 0.001). High prevalence of overweight, diabetes and low HDL were observed in third tertile of LI compared with lower tertiles. Individuals in the second tertile of LI had significantly higher prevalence of high LDL compared with lowest tertile (*P* = 0.02; Table 3). For adjustment of the possible confounders (e.g. sex, job, weight, region, education, smoking, energy, carbohydrate and protein) in linear regression analysis a positive association between LI and BMI (β = 0.17; *P* = 0.04), WC (β = 0.18; *P* = 0.01) and SBP (β = 0.27; *P* < 0.01; Table 4) was revealed. Dietary LI was positively associated with individual SFAs, including 4:0, 6:0, 8:0, 10:0, 12:0, 14:0, 15:0, 16:0, 17:0, 18:0, 20:0, 22:0, and 24:0 and inversely correlated with all polyunsaturated fatty acids (PUFAs), including 18:2 n-6 c و18:3 n-6, 18:4 n-6, 18:3 (n-3), 20:5 (n-3) and 22:6 n-3 (Table 5). A positive association between dietary LI and MUFA, including 14:1n-5, 16:1n-7, and 18:1n-9 and 24:1n-9, except 20:1n-9 was also observed. Also LI was positively correlated with total TFA 18:1, 16:1 and 18:2.

**Table 1:** The comparison of baseline characteristics by tertiles of dietary LI

Characteristics	Total (***N*** = 504)	LI tertiles			***P***-value
		1st tertile (***N*** = 166)	2nd tertile (***N*** = 167)	3rd tertile (***N*** = 171)	
Dietary LI ^a^ [mEq/day]	34.99 (6.91)	27.07 (4.57)	35.58 (1.75)	41.82 (3.11)	**< 0.001**
**Demographic variables**					
Age, years ^b^	42.53 (12.03)	42.64 (11.73)	42.68 (11.49)	42.29 (12.84)	0.94
Sex, *n* (%)					
Male	203 (44.2)	47 (31.1)	64 (42.1)	92 (59)	**0.01**
Female	256 (55.8)	104 (68.9)	88 (57.9)	64 (41)	
Household size	3.72 (1.03)	3.59 (1.01)	3.76 (0.97)	3.79 (1.1)	0.26
Occupation, *n* (%)					
Employee	32 (6.4)	8 (1.6)	11 (2.2)	13 (2.6)	**0.01**
Self-employment	141 (28.3)	29 (5.8)	48 (9.6)	64 (12.8)	
Family caregiver job, *n* (%)					
Employee	67 (13.4)	22 (4.4)	23 (4.6)	22 (4.4)	0.94
Self-employment	301 (60.3)	97 (19.4)	97 (19.4)	107 (21.4)	
Marital status, *n* (%)					
Single	32(6.4)	11 (2.2)	6 (1.2)	15 (3)	0.17
Married	470 (93.6)	151 (30.1)	159 (31.7)	160 (31.9)	
Region, *n* (%)					
Tabriz	237(47)	91(55.8)	83 (50)	63 (36)	**< 0.001**
Other	267 (53)	72 (44.2)	83 (50)	112 (64)	
Education, *n* (%)					
Illiterate	71 (14.1)	24 (4.8)	21 (4.2)	26 (5.2)	**< 0.001**
Diploma or Under diploma	352 (70)	98 (19.5)	121 (24.1)	133 (26.4)	
academic	80 (15.9)	40 (8)	24 (4.8)	16 (3.2)	
PA (MET-h/week) ^a^	9.8 (16.2)	11.5 (17.7)	9.5 (15.8)	8.3 (15.1)	0.2
Smoking, *n* (%)					
Current smoker	47 (9.4)	6 (1.2)	19 (3.8)	22 (4.4)	**0.03**
Never-smoker	447 (89.6)	152 (30.5)	145 (29.1)	150 (30.1)	
**Anthropometric variables [mean (SD)]**					
Weight (kg)	73.57 (13.31)	72.03 (12.74)	72.47 (12.49)	76.11 (14.28)	**< 0.001**
Height (cm)	163.44 (9.93)	161.86 (8.47)	163.23 (9.36)	165.13 (11.39)	**0.01**
BMI, kg/m^2 a^	27.64 (5.43)	27.45 (4.56)	27.29 (4.86)	28.18 (6.59)	0.2
WC (cm)	91.37 (12.93)	88.65 (15.03)	92.19 (11.94)	93.11 (11.3)	**< 0.001**
HC (cm)	104.27 (11.38)	105.42 (10.97)	103.76 (9.96)	103.69 (12.87)	
**Blood Pressure**					
SBP (mm Hg)	123.35 (17.19)	118.89 (17.46)	120.03 (15.42)	130.52 (16.24)	**< 0.001**
DBP (mm Hg)	78.33 (12.39)	79.8(12.16)	78.11 (11.45)	77.2 (13.36)	0.16
**Dietary factors**					
Carbohydrates (%)	56.5 (12.5)	58.6 (11.2)	56.4 (12.6)	54.5 (13.7)	**0.05**
Protein (%)	14.8 (4.4)	14.5 (3.4)	14.8 (4.2)	15 (5.5)	**0.03**
Fat (%)	29 (5.5)	26.9 (4.3)	28.8 (5.4)	30.5 (6.8)	**0.03**
Total energy ^a^	3454 (1420)	2755.2 (1232.5)	3211 (1022.2)	4381 (1454)	**< 0.001**

*LI* lipophilic index, *MET* metabolic equivalent; *BMI* body mass index, *WC* waist circumference; *HC* hip circumference; *SBP* systolic blood pressure; *DBP* diastolic blood pressure; *SD* standard deviation ^a^ Median (IQR) of continuous variables; ^b^ Mean (SD) of continuous variables. The bold values represents the significant threshold. The Kruskall-Wallis test or ANOVA are used for obtaining the results

**Table 2:** The mean (SD) values of metabolic biochemical variables by tertiles of dietary LI

Parameter [mean (SD)]	Total		LI tertiles						P _**trend**_
			1st tertile (***N*** = 166)		2nd tertile (***N*** = 167)		3rd tertile (***N*** = 171)		
	Mean	SD	Mean	SD	Mean	SD	Mean	SD	
**FBS (mg/dl)**	91.18	30.34	87	17.29	93.79	37.12	92.31	31.77	0.2
**LDL (mg/dl)**	89.02	23.52	82.52	20.51	91.8	29.72	92.07	17.45	**< 0.001**
**HDL (mg/dl)**	42.74	9.39	45.21	8.94	42.43	10.64	40.84	7.98	**< 0.001**
**TG (mg/dl)**	167.31	107.88	155.71	99.31	175.3	113.62	169.96	109.52	0.3
**TC (mg/dl)**	181.12	40.69	185.13	40.48	177	37.39	181.6	43.84	0.3
**AIP**	0.05	0.06	0.04	0.01	0.06	0.1	0.05	0	0.1
**LDL:HDL ratio**	2.45	2.98	1.99	0.51	2.92	4.8	2.33	0.52	0.1
**ALT (U/l)**	20.83	19.33	18.28	8.54	21.02	11.98	22.94	29.45	0.1
**AST (U/l)**	22.05	7.84	20.82	5.99	22.86	9.49	22.34	7.39	0.1

*SD* standard deviation; *FBS* fasting blood sugar; *LDL* low-density lipoprotein; *HDL* high-density lipoprotein; *TG* triglyceride; *TC* total cholesterol; *AIP* AthErogenic index of plasma; *ALT* alanine amino transferase; *AST* aspartate aminotransferase. The bold values represents the significant threshold. The *P*-values are obtained from Cochran-Armitage test

**Table 3:** The comparison of metabolic phenotypes by tertiles of dietary LI

Parameters (%)	Total (N = 504)	LI tertiles			P-value
		1st tertile (***N*** = 166)	2nd tertile (***N*** = 167)	3rd tertile (***N*** = 171)	
Overweight	294 (68.7)	112 (63.9)	112 (64)	330 (65.5)	**0.03**
Diabetes	36 (7.2)	6 (1.2)	11 (2.2)	19 (3.8)	**0.03**
Heart attack	15 (3)	7 (1.4)	3 (0.6)	5 (1)	0.4
Low HDL	275 (79.7)	76 (22)	90 (26.1)	109 (31.6)	**< 0.001**
High LDL	17 (4.8)	2 (0.6)	11 (3.1)	4 (1.1)	**0.02**
High TG	151 (42.1)	40 (11.1)	56 (15.6)	55 (15.3)	0.2
High TC	102 (28.3)	38 (10.5)	29 (8)	35 (9.7)	0.19
*High WC*	91 (18.8)	24 (5)	34 (7)	33 (6.8)	0.35
High FBS	35 (9.7)	7 (1.9)	12 (3.3)	16 (4.4)	0.24
High blood pressure	59 (12.1)	15 (3.1)	20 (4.1)	24 (4.9)	0.46

*SD* standard deviation; *FBS* fasting blood sugar; *LDL* low-density lipoprotein; *HDL* high-density lipoprotein; *TG* triglyceride; *TC* total cholesterol; The bold values represents the significant threshold. The chi-square test is used for obtaining the results

**Table 4:** Multivariate associations between LI and metabolic profile

Parameter	Standard β coefficient	P-value
**BMI**	0.17	**0.04**
**WC**	0.18	**0.01**
**SBP**	0.27	**< 0.001**
**DBP**	0.43	0.31
**FBS**	0.04	0.34
**TG**	0.07	0.28
**HDL**	−0.12	0.1
**LDL**	0.44	0.51
**TC**	−0.06	0.39

*SD* standard deviation; *FBS* fasting blood sugar; *LDL* low-density lipoprotein; *HDL* high-density lipoprotein; *TG* triglyceride; *TC* total cholesterol; *AIP* Atherogenic index of plasma; *ALT* alanine amino transferase; *AST* aspartate aminotransferase. The bold values represents the significant threshold. The *P*-values are obtained from Cochran-Armitage test. The bold values represents the significant threshold. * The linear regression analysis adjusted for sex, job, weight, region, education, smocking, energy, carbohydrate and protein is used for obtaining the results

**Table 5:** Melting points for different fatty acids and correlations between individual fatty acids and the LI

Fatty acids	Melting point (°C)	Individual FA intake (g/day)	r
**SFAs**			
24:0	−7.9	0.37 (0.6)	**0.78**
6:0	−3.4	0.21 (0.1)	**0.81**
8:0	16.7	0.13 (0.0)	**0.82**
10:0	31.6	0.24 (0.1)	**0.77**
12:0	44.2	0.26 (0.1)	**0.83**
14:0	53.9	0.83 (0.6)	**0.87**
15:0	52.3	NA ^a^	NA
16:0	62.9	7.2 (6.6)	**0.92**
17:0	61.3	0.07 (0.1)	**0.82**
18:0	69.6	3.23 (2.4)	**0.89**
20:0	75.3	0.06 (0. 2)	**−0.66**
22:0	81.5	0.07 (0.1)	**−0.32**
24:0	87.75	0.02 (0.01)	**0.53**
**MUFAs**			
14:1 n-5c	−4.5	0.13 (0.1)	**0.72**
16:1 n-7c	0	1.4 (0. 5)	**0.67**
18:1 n-9c	16	19.2 (14.5)	**0.16**
20:1 n-9c	23.25	0.1 (0.1)	**−0.14**
22:1 n-11	33.35	NA	NA
24:1 n-9	42.8	0.03 (0.05)	**0.26**
**PUFAs**			
n-6			
18:2 n-6c	−5	9.94 (7.9)	**−0.68**
18:3 n-6c	−11.5	0.1 (0.06)	**−0.21**
18:4 n-6c	−57	0.13 (0.13)	**−0.69**
20:2 n-6c	NA	NA	NA
20:3 n-6c	NA	NA	NA
n-3			
18:3 n-3c	−11.5	0.01 (0.01)	**−0.18**
20:5 n-3c	−53.5	0.01 (0.01)	**−0.59**
22:5 n-3c	−54.1	NA	NA
22:6 n-3c	−44.15	0.05 (0.07)	**−0.19**
**TFAs**			
16:1 T	31	0.01 (0.01)	**0.64**
18:1 T	48.7	2.3 (2.1)	**0.62**
18:2 T	5.7	0.2 (0.03)	**0.21**

*MUFA* monounsaturated fatty acid, *PUFA* polyunsaturated fatty acid, *SFA* saturated fatty acid, *TFA* Trans fatty acid; *NA* not applicable. The bold values represents the significant threshold. The Pearson’s correlation test is used for obtaining the results

## Discussion

In the current community based study in Tabriz-Iran, we demonstrated that the novel dietary lipophilic index (LI) was associated with higher age, being male, lower educational attainment, higher obesity and SBP. Higher dietary LI is associated with less membrane fluidity and high melting points of fatty acids. Higher LI in men can be explained by the evidence of higher saturated fatty acid intake and more adherence to western diet among them [20, 21]. In addition, high LI and WC versus low BMI in male group may be attributed to an association between LI and where the fat is accumulated. Mostly, men have an apple shaped body. Furthermore, individuals with higher fat and protein intake were in higher tertile of LI which clearly indicate the effect of dietary fat intake in predisposing individuals to MetS risk factors.

Moreover, individuals in higher tertiles of LI had higher SBP, lower carbohydrate and higher protein and fat intake in their usual diet.

Moreover, higher LDL and lower HDL cholesterol concentrations were also associated with increased LI values. Prevalence of low HDL, high LDL, overweight, and diabetes were also increased with increasing the LI and some of these associations even remained after adjustment for the confounders.

The estimated LI was used to sum up the individual fatty acids’ intake in an index that could be used to determine the mean fluidity of fatty acids consumed in the diet. Higher melting points correlate with a higher number of carbon atoms and a higher rating of saturation. Also, melting points are increased in *trans* rather than *cis* configuration. The median LI values for this study population was 34.99 (6.91) mEq/day. A higher LI, which indicates less fluidity, might be associated with increased accumulation of plasma LDL and its consequent insulin resistance and MetS; also, since the LDL receptor is a cell-surface receptor of nucleated cells and since fatty acids can change the properties of membranes [22], greater fluidity may also decrease the concentration of cholesterol by increasing its secretion. Furthermore, cell membrane is sensitive to ambient cholesterol concentration and increased membrane cholesterol reduces membrane fluidity, which results in reduced membrane permeability and filtration and consequently reduces cell survival [23, 24].

Our findings addressing an inverse association between LI and serum HDL concentration that reduced serum HDL was associated with increased LI and the same result was achieved in Toledo E's study too [9]. Likewise, low LI is associated with increased levels of HDL particles and increased Lecithin-cholesterol acyl transferase activity (LCAT). LCAT activity is important in determining plasma HDL concentration because LCAT deficiency results in reduced HDL concentration. Studies found that fluidity and membrane structure are key regulators of LCAT activity [15]. It has generally been accepted that increased membrane fluidity affects the physical barrier to oxygen permeation [24].

Thus, increased membrane strength due to higher dietary LI values may also impair endothelial cell function by reduced oxygen release. Also the less membrane fluidity reduces cell migration and results in wound closure [25]. In our study, the prevalence of high blood pressure was also increased with increasing LI. Our study confirms the results of the study by Tsuda K which reported an inverse association between increased cell membrane fluidity and hypertension [26]. In hypertensive subjects, the cell membrane deforms due to changes in the membrane composition. Also reduced HDL values was observed in numerous hypertensive subjects with elevated plasma LDL, very low density lipoprotein (VLDL), hypertriglyceridemia, hypercholesterolemia and insulin resistance [27].

Membrane fluidity depends on the fatty acids that make up the membrane. Membrane fluidity affects membrane permeability, receptor function and enzyme activity. Therefore, fluidity plays an important role in the pathogenesis of various MetS-related diseases. Changes in cell membrane structure have been known to regulate the function of proteins involved in ion transport, signal transduction, calcium ion transport, regulation of intracellular PH and other cell-related activities. Membrane structure also plays an important role in regulating blood pressure [25].

With respect to beneficial effects of PUFAs on health, LI exhibited a positive relationship with SFA and TFA and a negative relationship with PUFA [9]. Large amounts of PUFA reduce liver triglyceride production, increase cholesterol secretion and liver oxidation capacity. So, it is predicted that PUFAs may reduce the risk of insulin resistance by increasing membrane surface receptors and increasing insulin entry into the cells [27, 28]. Unsaturated fatty acids increase insulin values by increasing insulin receptors in the cell membrane. In contrast, insulin binding does not change in cells with high saturated fatty acids in their membranes [29, 30]. There is also evidence that membrane fluidity is strongly related to insulin sensitivity [27, 29, 31].

The current population-based study for the first time revealed the role of the novel dietary lipophilic index in prediction of metabolic abnormalities and obesity in a sample of Iranian adults. So LI may provide a better capture of fatty acids’ dietary pattern intake for predicting MetS risk factors. Several possible limitations of the current study should also be addressed; first, in the current study, the amount of several dietary trans fatty acids were negligible and therefore made up only a small fraction of the dietary LI. Because of the important role of trans fatty acids in the pathogenesis of diseases, this might affect the association of LI with metabolic risk factors. Second, the amount of these fatty acids in foods and biological samples is very low. Third, it seems that the melting point does not necessarily determine the fluidity of the cell membrane [9, 15]. From all of the 49 fatty acids, we had no data about melting point of 12 fatty acids [9] and finally, due to the cross-sectional design of the current study, it is impossible to infer the causal associations.

## Conclusion

Results of the current study revealed a positive association between dietary LI and several components of MetS and dyslipidemia. This is the first study that evaluated the association between dietary LI and components of MetS and dyslipidemia. Further studies in other disease could better highlight the clinical application of this index in pathogenesis of the disease.

## Data Availability

All of the data are available with reasonable request from the corresponding author.

## Abbreviations

MetS: Metabolic syndrome
DLP: Dyslipidemia
LI: Lipophilic index
MUFA: Monounsaturated fatty acid
PUFA: Polyunsaturated fatty acid
SFA: Saturated fatty acid
TFA: Trans fatty acid
USDA: United States department of agriculture
AIP: Atherogenic index of plasm
BMI: Body mass index
WC: Waist circumference
FBS: Fasting blood sugar
TC: Total cholesterol
TG: Triglyceride
HDL: High density lipoprotein cholesterol
LDL: Low density lipoprotein cholesterol
ALT: Alanine aminotransferase
AST: Aspartate Aminotransferase
